# *Heterosigma akashiwo* in Patagonian Fjords: Genetics, Growth, Pigment Signature and Role of PUFA and ROS in Ichthyotoxicity

**DOI:** 10.3390/toxins14090577

**Published:** 2022-08-23

**Authors:** Ana Flores-Leñero, Valentina Vargas-Torres, Javier Paredes-Mella, Luis Norambuena, Gonzalo Fuenzalida, Kim Lee-Chang, Jorge I. Mardones

**Affiliations:** 1 Centro de Estudios de Algas Nocivas (CREAN), Instituto de Fomento Pesquero (IFOP), Puerto Montt 5501679, Chile; 2 Instituto de Ingeniería Biológica y Médica (IIBM), Pontificia Universidad Católica de Chile, Santiago 8331150, Chile; 3 CAICAI Foundation, Puerto Varas 5550000, Chile; 4 Departamento de Ciencias Básicas, Facultad de Ciencias, Universidad Santo Tomás, Buena Vecindad #91, Puerto Montt 5504749, Chile; 5 CSIRO Ocean and Atmosphere, G.P.O. Box 1538, Hobart, TAS 7001, Australia; 6 Centro de Investigación en Dinámica de Ecosistemas Marinos de Altas Latitudes (IDEAL), Valdivia 5110566, Chile

**Keywords:** harmful algal blooms (HABs), raphidophytes, reactive oxygen species, fatty acids, salmon farming, Chile

## Abstract

*Heterosigma akashiwo* is the only raphidophyte described for Chilean waters. A recent 2021 fish-killing bloom event of this raphidophyte ignited scientific research, but the ichthyotoxic mechanism and environmental conditions that promote its growth are still unclear. This is the first study confirming the occurrence of *H. akashiwo* in Chilean waters on the basis of the region D1/D2 of the 28S ribosomal gene. The pigment signature of the CREAN_HA03 strain revealed chlorophyll-a, fucoxanthin, and violaxanthin as the most abundant pigments, but profiles were variable depending on culture and field conditions. A factorial temperature–salinity growth experiment showed a maximal growth rate of 0.48 d^−1^ at 17 °C and 35 in salinity, but reached a maximal cell abundance of ~50,000 cells mL^−1^ at 12 °C and 25 in salinity. The fatty acid profile included high levels of saturated (16:0) and polyunsaturated (18:4 ω3; 20:5 ω3) fatty acids, but superoxide production in this strain was low (~0.3 pmol O^2–^ cell^−1^ h^−1^). The RTgill-W1 bioassay showed that the *H. akashiwo* strain was cytotoxic only at high cell concentrations (>47,000 cells mL^−1^) and after cell rupture. In conclusion, salmon mortality during *H. akashiwo* bloom events in Patagonian fjords is likely explained by the high production of long-chain PUFAs at high cell densities, but only in the presence of high ROS production.

## 1. Introduction

*Heterosigma akashiwo* is a small raphidophyte that is widely distributed in coastal ecosystems and is well-known as a driver of brown tides in many parts of the world. This species is a marine phytoflagellate that is described as an eurythermal and euryhaline organism, meaning that it can tolerate broad ranges of temperatures from 11 to 24 °C [1,2,3] and salinity from 5 to 35 [2,4]. Along with this, *Heterosigma akashiwo* has the ability to form resting cysts [5] when exposed to adverse conditions that coincide with seasonal differences in light, temperature, and nutrients present in temperate climates [6,7]. Another adaptation mechanism is the large quantity of pigments with photoprotective and antioxidant functions, enabling this microalga to live under high irradiance. *Heterosigma akashiwo’*s pigment profile contains Chl a, Chl c1, Chl c2, beta-carotene, and carotenoids fucoxanthin and violaxanthin as its pigment markers [2]. These characteristics possibly give *H. akashiwo* a competitive advantage over other organisms, allowing for it to colonize coastal ecosystems, such as bays and estuaries [4].

Despite the wide range of abiotic conditions in which this species can live, strains of *H. akashiwo* extracted from blooms in different locations have shown surprisingly low genetic variability. Engesmo et al. [8] demonstrated that the rDNA of *H. akashiwo* shows signs of segregation consistent with geographical origin, differentiating at least two polymorphic positions within small-subunit (18S) rDNA, one specific for strains extracted from Australia or New Zealand, and the other from the Atlantic coast of North America. However, in the same study, *H. akashiwo* strains displayed low genetic variability in the ITS region, which is considered to be the most variable rDNA region.

The blooms of *H. akashiwo* have been implicated in fish-killing events in several coastal areas, including Canada, Japan, New Zealand and Chile [9], and have been associated with economic losses to the aquacultural industry in those countries [10]. In Chile, the first *H. akashiwo* salmon-killing event was reported in September 1988 in the Los Lagos region (Reloncavi sound, Hornopiren and Chiloe Island), where the alga’s maximal cell density reached 100,000 cells mL^−1^ [3]. In April 2021, a massive *H. akashiwo* bloom occurred in the Comau fjord, Northern Patagonia, with cell abundances that ranged between 70,000 and 210,000 cells mL^−1^, causing a mass mortality of farmed salmon [11]. The historical monitoring of the Chilean *H. akashiwo* has shown its occurrence at temperatures ranging from 11.2 to 17.3 °C, and salinities from 11.4 to 33.3 [3], and ichthyotoxicity linked to high cell densities. A recently performed cell line bioassay analysis on the Chilean *H. akashiwo* has supported this hypothesis, with its ichthyotoxicity found to be low at concentrations of 2000 cells mL^−1^ [12,13].

The Raphidophyceae class is well-known for being responsible for massive fish mortalities, and while the cytotoxic compounds produced by *H. akashiwo* are still not well-understood, some possible ichthyotoxic mechanisms have been hypothesized. One mechanism involves the production of a putative neurotoxin with a similar effect to brevetoxins; however, this compound is not structurally related to the brevetoxin group, so the mode of action on fish remains unknown [14,15]. Another potential mechanism is the production of haemolytic compounds that can affect different tissues and organs in fish. These metabolites, along with the production of reactive oxygen species (ROS) such as hydrogen peroxide, hydroxyl radicals, and superoxide can produce severe injuries to fish gill tissues such as cell necrosis, epithelial lifting, and the alteration of chloride cells [16]. These injuries produce excessive mucus secretion that can cover the fish gills and cause asphyxiation [17]. However, ROS on their own may not be the main cause of ichthyotoxicity in this species [16]. Some authors proposed that the toxicity could be related to the presence of high concentrations of polyunsaturated fatty acids (PUFAs) [18]. The production of mucus or extracellular polymeric substances, such as polysaccharide–protein complexes (A–PCs), was also proposed as a toxic mechanism in *H. akashiwo* [19].

The main objective of this study is to define the phylogenetic position, pigment signature, superoxide production, fatty acid profile, and potential toxic effects of a Chilean *H. akashiwo* strain, and the environmental conditions that might promote its growth in the Patagonian fjords.

## 2. Results

### 2.1. Molecular Identification

**Figure 1:**
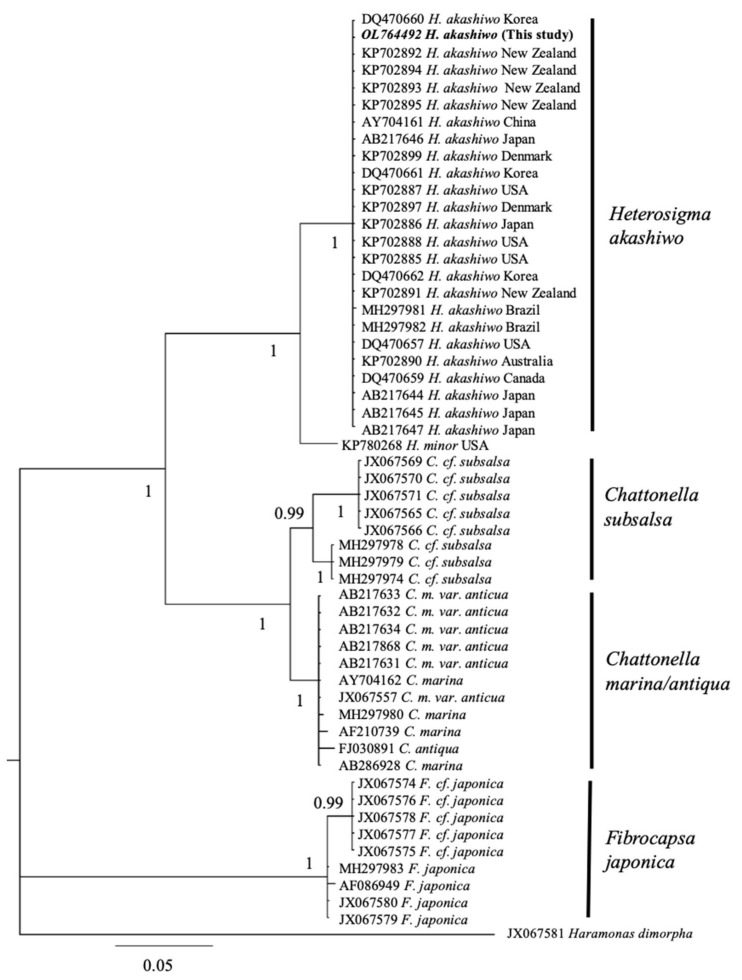
Phylogeny of the large-subunit (LSU rDNA) sequence of the Chilean *H. akashiwo* strain and related species. In bold is the Chilean CREAN sequence analysed in this study.

The Bayesian phylogenetic and maximal likelihood analysis of the LSU D1–D2 region showed that the Chilean *H. akashiwo* (CREAN_HA03) sequence is in a monophyletic clade that comprises only *H. akashiwo* sequences, supported by one probability value (Figure 1). The monophyletic clade shows that this sequence of *Heterosigma* has a genetic distance of 0.00 against the sequences reported in Japan, Australia, EEUU, Demark, China, and New Zealand. In addition, the genetic distance with strains from Brazil, Canada, Korea, and New Zealand was 0.001, and the distance with H. minor from the USA resulted in a value of 0.064.

### 2.2. Pigments Signature

**Figure 2:**
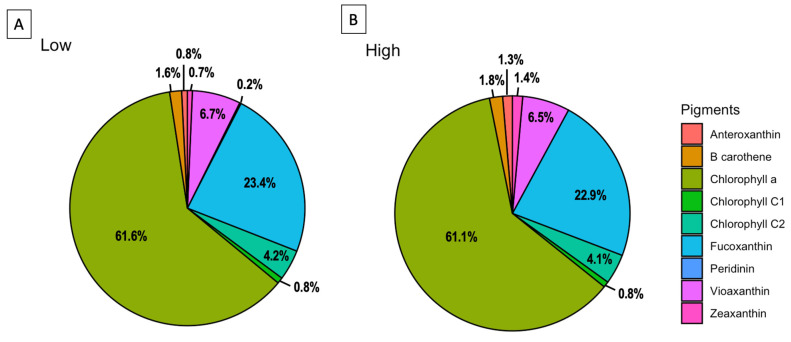
Pigment profiles of the CREAN_HA03 strain cultured at two different light conditions. (**A**) Low-light (LL) treatment at 50 μmol photon m^−2^ s^−1^; (**B**) high-light (HL) treatment at 150 μmol photon m^−2^ s^−1^. The chart shows the percentage of each pigment over the total pigment content.

The pigment analysis of the *H. akashiwo* culture (CREAN_HA03), grown under the two light conditions of 50 (low light, LL) and 150 μmol photon m^−2^ s^−1^ (high light, HL), shows a similar pattern in pigment concentrations. The most abundant pigment was chlorophyll-a with a concentration of 8.9 at LL and 7.16 pg cell^−1^ at HL exposure (62% and 61% of the total pigments, respectively). The most abundant carotenoids at the LL and HL treatments were as follows: fucoxanthin with 3.38 and 2.68 pg cell^−1^ (23% of the total pigments in both samples), and violaxanthin with 0.96 and 0.76 pg cell^−1^ (7% of the total pigments in both samples), followed by chlorophyll c2, β-carotene, antheraxanthin, zeaxanthin, chlorophyll c1 and peridinin (Figure 2).

The HPLC pigment profiles of a bloom sample collected during the 2021 *H. akashiwo* event, and three profiles from a cultured strain (CREAN_HA03) measured at two different periods of time (one in 2020 at 100 μmol photon m^−2^ s^−1^, and two in 2022 at 50 and 150 μmol photon m^−2^ s^−1^) are presented in Table 1. The pigment distribution of the 2021 bloom sample shows a similar pattern compared with the CREAN_03 strain measured in 2020 but differed in pigment content (%) with the same strain measured in 2022. Ratios of Fuco:Chl a for the 2021 field sample and CREAN_HA03 strain measured in 2020 were 4.98 and 3.29, respectively. The Fuco:Chl a ratios of the CREAN_HA03 strain measured in 2022 showed lower values of 0.38 and 0.37 (50 and 150 μmol photon m^−2^ s^−1^ light, respectively).

### 2.3. Growth Rate and Cell Density

**Figure 3:**
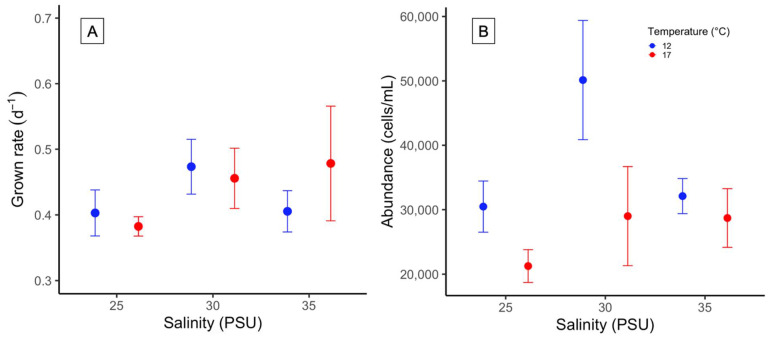
In vitro (**A**) growth rate (d^−1^) and (**B**) maximal cell abundance (cells mL^−1^) of *H. akashiwo* at two temperatures (12 and 17 °C), and at three different salinities (25, 30 and 35). Symbols represent the mean and error bars the standard error from triplicate measurements.

The response of the Chilean H. akashiwo (CREAN_HA03) to different salinity and temperature conditions was not significantly different in terms of growth rate (μ; ANOVA, *p* > 0.05). The μ ranged between 0.38 ± 0.03 d^−^^1^ and 0.48 ± 0.15 d^−^^1^. The lowest µ_max_ was reached at 25 in salinity at 17 °C, and the highest at 35 in salinity at 17 °C, respectively (Figure 3A). Temperature significantly affected the maximal cell abundance on Day 19, reaching a density of 50,000 cells mL^−^^1^ at 12 °C, and 30 in salinity (ANOVA, *p* = 0.03, Figure 3B).

### 2.4. Fatty Acid Profile and Superoxide Production

The fatty acid composition of *H. akashiwo* strain CREAN_HA03 was dominated by polyunsaturated fatty acids (PUFAs) with a 40.4%, followed by saturated fatty acids (SFAs), and monounsaturated fatty acids (MUFAs) with 30.7% and 22.2%, respectively. The main fatty acids in relative abundance were, in decreasing order, palmitic acid (16:0 PA; 20.94%), EPA (20:5ω3; 13.04%), and stearidonic acid (18:4ω3; 10,44%) (Table 2).

Whole cells of H. akashiwo CREAN_HA03 showed similar rates of superoxide anion production (0.3075 ± 0.0299 pmol O^2−^ cell^−1^ h^−1^) compared to those of lysed cells (0.2925 ± 0.0126 pmol O^2−^ cell^−1^ h^−1^) (Table 3).

### 2.5. Intra- and Extracellular Ichthyotoxicity

**Figure 4:**
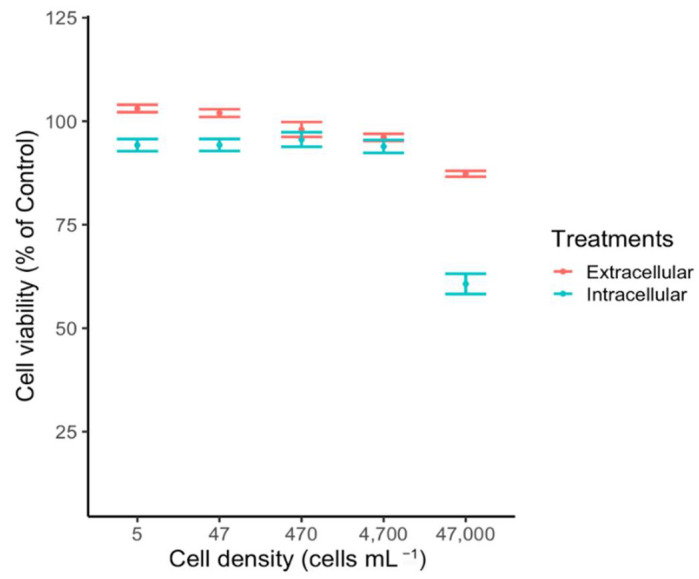
Effect of *H. akashiwo* intracellular content (blue) and extracellular exudates (red) at five different cell abundances (47,000; 4700; 470; 47; 5 cells mL^−1^) on gill cell viability (% of control). Circles represent the mean and error bars the standard error of cell viability from quadruplicate measurements.

Lysed (intracellular) and supernatant (extracellular) treatments at different cell abundances of H. akashiwo exposed against the RTgill-W1 cell line showed distinct effects only at high cell abundances. The highest cytotoxic effect was measured in the intracellular treatment at 47,000 cells mL^−^^1^, significantly decreasing gill cell viability down to 61% of control (ANOVA, *p* < 0.05, Figure 4). Extracellular treatment at 47,000 cells mL^−^^1^ only reduced the viability down to 90% of control. In either treatment, low densities did not have a significant effect against the Rtgill-W1 cell line (Figure 4).

## 3. Discussion

*Heterosigma akashiwo* is the only raphidophyte species that has been reported in Chilean waters producing deleterious effects on salmon farming activities. The massive bloom event of this species that occurred in austral summer–fall 2021 in the Comau fjord, ignited new research attention on this poorly studied species in the Chilean coast. In this study, we assessed the phylogenetic position and in vitro characteristics of a Chilean *H. akashiwo* strain with special emphasis on its ichthyotoxic potency.

To our knowledge, this is the first formal genetic identification of *H. akashiwo* in Chilean waters. Previous studies carried out in Chile only identified this raphidophyte on the basis of morphological characteristics [3,12,13,17]. In terms of genetic variability, the Chilean 28S rDNA sequence showed low intraspecific genetic variability against worldwide distributed *H. akashiwo* genetic sequences [20]. *Heterosigma akashiwo* sequences formed a monophyletic clade [8] expressing low genetic divergence regardless of geographical patterns [21,22]. As no further studies on Chilean *H. akashiwo* have focused on the intraspecific variation in the physiological response of local strains, the presence of different ecotypes is still unknown. A suggested direct association between chloroplast gene signatures and ecophysiological diversity allows, for instance, for an avenue for tracking different *H. akashiwo* populations from the west coast of North America [23]. The examination of selected regions within the chloroplast genome of additional Chilean strains may allow for the detection of strain-specific sequences and ecotypes that explain the ability of this species to form persistent blooms, as observed in 2021 in the highly variable Patagonian fjords ecosystem.

This study shows that the Chilean *H. akashiwo* has a flexible pigment signature in response to environmental conditions. This is in line with the well-known capacity of phytoplankton cells to modify their pigment content and ratios under different light and nutrient conditions [24]. Our data show that the most abundant secondary pigments in the CREAN_HA03 strain were fucoxanthin and violaxanthin. Other authors also found fucoxanthin to be the most abundant carotenoid in *H. akashiwo* strains from other coastal areas [2] and among others Chilean strains [17]. Under high irradiance conditions, microalgal cells increase photoprotective carotenoids, such as fucoxanthin, violaxanthin, and antheraxanthin, to prevent photo-oxidative damage [25,26]. In fact, a high fucoxanthin concentration was detected in field samples from the 2021 *H. akashiwo* bloom event at irradiances as high as 1430 μmol photon m^−2^ s^−1^, with a Fuco/Chl-a ratio of 4.98 (Table 1). However, a similar pigment proportion was found in the CREAN_HA03 strain one year after its isolation (harvested in 2020) at a low irradiance of 100 μmol photon m^−2^ s^−1^ with a Fuco/Chl-a ratio of 3.39, suggesting that pigment signatures could be conservative after cell isolation and culture establishment. This assertion was further denied after assessing the pigment signature of the same *H. akashiwo* strain cultured at 50 and 150 μmol photon m^−2^ s^−1^, which decreased fucoxanthin and increased Chl-a concentrations, reaching Fuco/Chl-a ratios of 0.38 and 0.37, respectively. Some authors suggested that Fuco/Chl-a ratios do not always dependent on light intensities [2], as factors such as the availability of nutrients or external stressor conditions can induce carotenoid production or degradation by microalgae [26,27]. For instance, (i) the mixotrophic behaviour observed in microalgal species (e.g., *H. akashiwo*) induces fucoxanthin accumulation [28,29], and (ii) the diel vertical migration (DVM) observed in *H. akashiwo* may also produce variations of pigment/Chl-a ratios with depth in the water column as an ecophysiological response of photoprotective and light harvesting functions. Thus, caution must be taken, for instance, when using pigment signatures for chemotaxonomic purposes during *H. akashiwo* blooms.

In this study, growth rates obtained from the Chilean *H. akashiwo* strain that ranged between 0.38 ± 0.03 and 0.48 d^−^^1^ were consistent with growth rate values reported from experiments carried out under similar environmental conditions (values between 0.319 and 0.455 d^−^^1^) [2,4]. In contrast, recent studies have reported higher growth rate values and enormous cell abundances from other Chilean *H. akashiwo* strains (1,000,000 cells mL^−^^1^) that did not relate with natural field cell densities reported from bloom events that occurred in southern Chile (max. 100,000 cells mL^−^^1^) [13,17]. Despite differing results being able to respond to the intraspecific variation of the strains, attention on cell count methods used in these studies must be taken. As *H. akashiwo* cells are quite small and extremely fragile, the Uthermöhl and Sedgewick-Rafter chambers, strong cell fixatives (e.g., Lugol), and long sample storage periods are not recommended. The use of haematocytometers (e.g., Fuchs-Rosenthal and Neubauer chambers), low concentrations of glutaraldehyde (1% final concentration), and immediate sample analysis may improve cell count results. On the other hand, no differences were observed in the *H. akashiwo* (CREAN_HA03 strain) growth response under different salinity and temperature treatments, stressing its euryhaline and eurythermal ability to grow in the environmentally complex Patagonian waters. These results are in line with most of the global studies reporting the positive growth of this raphidophyte species at salinities between 5 and 35 [2,27], and with the detection of this species in Patagonian estuarine waters in a range of salinity between 11 and 33 [3]. In vitro cultures of the Chilean CCM-UdeC 225 strain coincided with better growth performance at low salinity levels of 15 and 20 [13,17]. The main differences observed in growth rate and maximal cell abundances between the CCM-UdeC 225 and the CREAN_HA03 strains might correspond to their contrasting origin of offshore vs. fjord environments, respectively.

This study shows that the Chilean CREAN_HA03 strain is lightly toxic only at high cell densities. This finding goes against previous studies that state that *H. akashiwo* is cytotoxic at low algal concentrations [13,17]. This fact might be due to the different methods used to determine the in vitro cytotoxic potency of the Chilean *H. akashiwo*. For instance, Gómez et al. [17] studied the toxic effects produced by a strain (CCM-UdeC 225) of this raphidophyte on *Artemia salina* larvae, reporting a maximal effective concentration (EC50) of 1937 cells mL^−^^1^. Sandoval-Sanhueza et al. [13], using the same *H. akashiwo* strain, observed 100% reduction in the viability of cell line CHSE-214 derived from embryos of Chinook salmon. Other studies using Chilean and Australian *H. akashiwo* strains [12,18] reported mildly toxic effects against the RTgill-W1 fish gill cell line. Low toxic effects are more in line with measurements carried out in the field during the 2021 *H. akashiwo* bloom event, where salmon mortality was low (6000 tons) compared with the extreme raphidophyte cell abundances reported during weeks (>70,000 cell mL^−^^1^; IFOP, unpublished data). A plausible explanation for the differences in the cytotoxic potency reported for the Chilean *H. akashiwo* strains is their geographical origin. While the CCM-UdeC 225 strain was isolated from an estuarine area with strong oceanic influence, the CREAN_HA03 strain was isolated from the Comau fjord, which is characterized by intensive salmon farming activities. On the other hand, it is also possible that the use of different methods for assessing ichthyotoxicity among the Chilean strains might have led to different conclusions. It is important to mention that an international colloquium of HAB experts held in Chile in 2019 resolved that the RTgill-W1 bioassay is the most suitable methodology to determine ichthyotoxicity so far.

Gill cell assay experiments using the CREAN_HA03 strain showed that exudates released into the media were less toxic than intracellular compounds. This observation is in contrast with results obtained by Sandoval-Sanhueza et al. [13], and Gómez et al. [17], who observed that intracellular and extracellular compounds produced by CCM-UdeC 225 exerted similar toxic effects. The production of toxic extracellular compounds might be related with prey capture when *H. akashiwo* switches to a mixotrophic behaviour [30].

This study assesses the first lipid composition and ROS production (as superoxide anion) of a Chilean *H. akashiwo* strain. The CREAN_HA03 strain showed high amounts of palmitic acid (PA 16:0; 20.94%), followed by the PUFA eicosapentaenoic acid (EPA 20:5ω3; 13.04%), and stearidonic acid (18:4ω3; 10,44%). Despite saturated fatty acids such as palmitic acid, and PUFAs with large carbon chains and double bounds that are toxic to gill cells [18], toxicity is exerted when present only at high concentrations. The high concentration of PUFAs in the Chilean CREAN_HA03 strain (40% of all FA) is in line with the lipid profile of an Australian *H. akashiwo* strain (HABG01) (PUFAs 52%) [18]. On the other hand, superoxide production by the CREAN_HA03 strain was low (~0.3 pmol O^2-^ cell^−^^1^ h^−^^1^) compared with that of other ichthyotoxic species such as *K. selliformis* and *A. catenella* that are associated with fish-kill events in Chilean waters [31,32]. The low O_2_^–^ production by the Chilean CREAN_HA03 suggests that this reactive molecule does not play an important role in enhancing the toxicity of PUFAs during *H. akashiwo* bloom events in southern Chile. This result supports the low ichthyotoxicity observed during the 2021 bloom event at the Comau fjord, Los Lagos region. Further studies should explore other toxic mechanisms, such as the production of mucus or extracellular polymeric substances, for the advanced understanding of fish-killing events attributed to *H. akashiwo* in the south of Chile.

In summary, this is the first study (1) genetically confirming the presence of *H. akashiwo* in Chilean waters, and reporting (2) ROS production and (3) a lipid profile from a Chilean strain. The Chilean CREAN_HA03 strain showed euryhaline and eurythermal behaviour, a characteristic that is commonly described for other globally distributed strains. The high variability in fucoxanthin observed in the Chilean *H. akashiwo* was not directly associated with light intensity, suggesting an important role of other chemical (e.g., nutrients), and biological (e.g., mixotrophy) factors in its synthesis. The high production of saturated and polyunsaturated fatty acids by Chilean *H. akashiwo* may contribute to some extent to the observed ichthyotoxicity during bloom events. However, the low superoxide anion production suggests further research on other ichthyotoxic mechanism in this raphidophyte besides the ROS/PUFA synergistic reaction.

## 4. Materials and Methods

### 4.1. Microalgal Culture Conditions

One monoclonal culture of *H. akashiwo* (CREAN_HA03 strain) was isolated from the Comau fjord in 2019 (42°22.166′ S, 72°27.30′ W; Los Lagos region). The CREAN_HA03 strain was maintained in culture at the CREAN-IFOP algal collection in Puerto Montt, Chile. Nonaxenic cultures were grown in L1 medium at 15 °C in sterile filtered (0.22 μm), in seawater at 33 of salinity at 100 μmol photon m^−2^ s^−1^ (cool white fluorescent lamps) under an 18:6 h light:dark cycle.

### 4.2. DNA Extraction, Amplification, Sequencing, and Phylogeny

The CREAN_HA03 culture was centrifugated for 10 min at 1500× *g* at room temperature, and the supernatant was discarded. The cell pellets were then incubated in 1 mL cetyltrimethylammonium bromide (CTAB) and 10 μL proteinase K (10 mg mL^−1^) for 1 h at 65 °C. The region D1/D2 of the 28S ribosomal gene was amplified using D1–D2 primers [33,34]. The PCR protocol started with an initial denaturation step for 1 min at 95 °C, and then 35 cycles for 1 min at 95 °C, 56 °C for 1 min, 72 °C for 1 min, followed by a 7 min extension at 72 °C. The visualisation of PCR products was carried out on a 1.5% agarose gel, then a purification step was performed using the Illustra GFX PCR DNA and gel band purification kit (GE Healthcare Chicago, Illinois, USA), and the sequencing of ribosomal regions was carried out in Macrogen Sequencing Facility (Macrogen^®^, South Korea). Phylogenetic reconstruction was carried out on the basis of available sequences in GenBank using an alignment of 920 bp. Maximal likelihood analysis (ML) was assessed using PhyML Version 3.0 software [35], and appropriate models for sequence evolution (LSU: GTR + I) were identified using jModeltest [36]. The likelihood ratio test (aLRT) was used to estimate the node reliability [37] and bootstrap analysis (1000 replicates). Lastly, Bayesian analyses were conducted using MrBayes V3.1.2 under the appropriate model (GTR + I) [38].

### 4.3. HPLC Pigment Analysis

A field sample (200 mL) collected from the 2021 *H. akashiwo* bloom event at the Comau fjord, and three cultures (40 mL) of the CREAN_HA03 strain grown at three light levels (50, 100, and 150 μmol photon m^−2^ s^−1^) were used for pigment analysis. The field sample and the *H. akashiwo* cultures (in the exponential growth phase) were centrifuged at 3000 rpm for 20 min, and the cells collected on these pellets were subsequently extracted in 1.5 mL acetone (100%) after probe sonication (60 s) and soaking for 24 h at −20 °C. Photosynthetic pigments were measured using a Shimadzu high-performance liquid chromatograph (HPLC) with a quaternary LC-10AT pump, a Sil-10AF autosampler, DGU-14A degasser, and CBM-20A system controller as described by Sanz et al. [39]. Chromatographic separation was carried out using an ACE C18 PFP column of 150 × 4.6 mm, 3 μm particle size (Advanced Chromatography Technologies, Aberdeen, UK) at 40 °C. Mobile phase B was arranged with ethanol, and mobile phase A with methanol: 225 mM ammonium acetate (82:12 m *v*/*v*). The gradient was programmed at a flow rate of 1.0 mL min^−1^. Certified reference standards for chlorophyll a, peridinin, chlorophyllide a, chlorophyll c2, chlorophyll c1, fucoxanthin, violaxanthin, antheraxanthin, zeaxanthin, antheraxanthin, B-carotene were obtained from DHI (DHI Laboratory Products, Hoersholm, Denmark). Ammonium acetate, ethanol, and methanol were of HPLC gradient grade (Merck; Darmstadt, Germany).

### 4.4. Lipid Extraction and Analysis

*H. akashiwo* (CREAN_HA03 strain) samples were extracted using the modified Bligh and Dyer method with dichloromethane/methanol/water (1:2:0.8, *v*/*v*/*v*) as described by Mooney et al. [40]. The detailed description of lipid extraction and analysis was provided by Mardones et al. [31]. Briefly, the extracted lipids were transmethylated, concentrated using nitrogen gas, and analysed by gas chromatography using an Agilent Technologies 7890 N GC (Palo Alto, Santa Clara, CA, USA) equipped with an Equity-1 crosslinked methyl silicone-fused silica capillary column (15 m × 0.1 mm i.d.), and an FID. The FAME identification was assessed by comparing with retention times of laboratory standards. Gas chromatography–mass spectrometry (GC–MS) analyses were carried out using a Finnigan Thermoquest GCQ GC–MS, and using Thermoquest Xcalibur software (Austin, TX, USA).

### 4.5. Production of Superoxide Anion by H. akashiwo

The lysed cultures and intact algal cells of the CREAN_HA03 strain were assessed for superoxide anion production using the method described by Godrant et al. [41]. Briefly, the CREAN_HA03 strain cultured at 17 °C at 100 mmol photon m^2^ s^−1^ (cool white fluorescent lamps) under a 12:12 h light:dark cycle was harvested at exponential growth. From the harvested sample, two treatments of intact and lysed cells were set up. The lysed cell suspension was prepared with the sonication of samples for 2 min at an amplitude of 10 μm peak to peak at 17 °C. Using a 96-well microplate, 270 mL of the *H. akashiwo* culture was mixed with 3 mL of xanthine (X7375, Sigma, St. Louis, MO, USA) at 5 mM. The blank correction was set using 3 mL of superoxide dismutase (S7571, Sigma) at 5 kU L^−1^. A standard curve was established using xanthine oxidase (X1875, Sigma) at 0.7, 0.4, and 0.1 U L^−1^. After adding 5 mL of MCLA, 6-(4-methoxyphenyl)-2-methyl-3,7dihydroimidazo [1,2-a]pyrazin-3(7H)-one hydrochloride (87787, Sigma) at 125 mM, luminescence was monitored for 20 min using a microplate reader (FLUOstar OPTIMA, BMG Labtech, 413-3350 Ortenberg, Germany).

### 4.6. RTgill-W1 Cell Line Maintenance

Cell line RTgill-W1 [42] was obtained from the American Type Culture Collection (CRL-2523, ATCC, Manassas, VA, USA). For culturing, gill cells were maintained in the dark at 19 °C in Leibovitz’s L-15 medium (L1518 Sigma) supplemented with an antibiotic–antimycotic solution (A5955, Sigma) containing amphotericin B (25 mg mL^−1^), streptomycin (10 mg mL^−1^), and penicillin (10,000 units mL^−1^), and 10% (*v*/*v*) fetal bovine serum (FBS, 12003C, Sigma), in 25 cm^2^ culture-treated flasks. TrypLE™ Express (Gibco™) was used to remove cells from the bottom of the flask. Subcultures were routinely set twice per week at a ratio of 1:2 with L-15 medium renewal.

### 4.7. Gill Cell Assay with H. akashiwo

Gill cell viability was assessed using conventional 96-well microplates according to Dorantes-Aranda et al. [43]. The CREAN_HA03 strain cultured at 15 °C and salinity of 27 was prepared at 5 different cell abundances (47,000; 4700; 470; 47; 5 cells mL^−1^) for experimental treatments. Cultures with confluent gill cells were trypsinized for detachment, counted using a haemocytometer, and adjusted to a concentration of 1–2 × 10^5^ cells mL^−1^. Subsequently, gill cells were seeded in quadruplicate in 96-well flat-bottom microplates (3860-096, Iwaki, Shizuoka, Japan), using a volume of 100 μL per well. After 48 h at 19 °C in the dark for gill cell attachment, the L-15 medium was discarded and exposed to 100 μL of the experimental dinoflagellate treatments. The extracellular treatment was set with the centrifugation of the *H. akashiwo* culture at 3500 rpm for 10 min and then diluted as needed. The intracellular treatment was prepared through the sonication of diluted samples for 2 min at an amplitude of 10 μm peak to peak at 17 °C and filtered using a syringe with a nylon filter (0.22 μm). The exposure of *H. akashiwo* extra- and intracellular compounds was performed for 2 h at 19 °C in the dark.

Gill cell viability was determined using an L-15/ex medium, a modified version of the L-15 medium, containing 5% of the indicator dye alamarBlue (DAL1025, Invitrogen, Waltham, MA, USA) [44]. The medium was added to all cell-seeded wells and incubated for 1 h in the dark [43]. Using a microplate reader (FLUOstar Omega, BMG Labtech 415-2871), the fluorescence of alamarBlue was detected with excitation and emission filters of 540 and 590 nm, respectively. The viability of gill cells was expressed as the response percentage of the treatments relative to the controls (% of control).

### 4.8. H. akashiwo Growth under Different Temperatures and Salinities

The *H. akashiwo* (CREAN_HA03 strain) experiments were carried out in triplicate using sterile flasks, each containing 45 mL of L1 culture medium inoculated with 300 cells mL^−1^. The growth rate and culture yield were studied using a crossed factorial design with 6 different conditions, obtained by combining three salinities (25, 30, and 35) and two temperatures (12 and 17 °C). These different salinities and temperature ranges are commonly found within the inner Patagonian fjords. To prevent modifications in the physiological response of *H. akashiwo* due to fluctuations in salinity and temperature, cultures were preacclimated to the required experimental conditions for a period of >20 days. The experiment was set for 19 days, and every 2–3 days, samples were collected and fixed with buffered glutaraldehyde. Cell abundance was assessed instantly after sampling under an inverted light microscope using a Fuch-Rosenthal chamber. The mean cell number obtained from the three replicates was used to estimate the growth rate *μ* (d^−1^) as follows:

$$\mu =\frac{\ln\left(c_{1}/c_{0}\right)}{t_{1}-t_{1}}$$

where *c*_0_ and *c*_1_ are the cell densities (cells mL^−1^) at the beginning (*t*_0_) and end (*t*_1_) of the incubation period (days), respectively.

### 4.9. Statistical Analysis

To assess the effect of environmental variables on growth and ichthyotoxicity of the Chilean *H. akashiwo* strain, analysis of variance (ANOVA) was performed. The normality and homogeneity of variances were assessed with the Shapiro–Wilk and Levene tests, respectively. An a posteriori Tukey test was performed to identify the differences between the treatments. All analyses were carried out using the software R v. 3.0.1 [45].

## Figures and Tables

**Table 1:** Comparison of pigment profiles measured in field and cultured *Heterosigma akashiwo* samples.

Pigments	Sample	Bloom *H. akashiwo*	CREAN_ HA03		
	Year	2021	2020	2022	
	Light Intensity	1430	100	50	150
Peridinin		ND	ND	0.20	ND
Chlorophyll C1		7.24	2.73	0.84	0.80
Chlorophyll C2		15.09	18.32	4.19	4.11
Fucoxanthin		48.78	48.43	23.41	22.84
Violaxanthin		14.73	9.77	6.67	6.77
Antheraxanthin		1.78	3.80	0.80	1.34
Zeaxanthin		1.60	2.25	0.68	1.34
Chlorophyll a		9.80	14.70	61.59	60.95
β-Carotene		ND	ND	1.63	1.84
Chlorophyllide a		0.98	0.00	ND	ND
Ratio Fuco/Chl a		4.98	3.29	0.38	0.37

Abbreviations: ND, not detected. Note: light intensity in μmol photon m^−2^ s^−1^.

**Table 2:** Fatty acid profile of the Chilean *Heterosigma akashiwo* (as % of total fatty acids).

Fatty Acid	Mean		SD
**SFA**			
13:0	0.02	±	0.02
14:0	5.47	±	0.08
15:0	0.83	±	0.04
16:0 PA	20.94	±	0.14
17:0	0.25	±	0.01
18:0	2.77	±	0.09
20:0	0.07	±	0
22:0	0.16	±	0.13
24:0	0.02	±	0.02
Hexadecenal	0.04	±	0.05
Octadecenal	0.15	±	0
**Branched**			
a15:0	0.01	±	0.01
i14:0	0.02	±	0.02
i15:0	0.63	±	0.01
i16:0	0.22	±	0.01
i17:0	0.03	±	0
i18:0	0.16	±	0.02
**MUFA**			
17:1ω8c + a17:0	0.15	±	0.03
14:01	0.06	±	0.02
16:1ω9c	0.33	±	0.29
16:1ω7c	8.60	±	0.09
16:1ω7t	0.02	±	0.03
16:1ω5c	2.54	±	0.02
16:1ω13t	1.05	±	0.91
18:1ω9c	1.36	±	0.04
18:1ω7c	7.09	±	0.09
18:1ω7t	0.05	±	0.05
18:1 a	0.10	±	0.08
18:1 b	0.04	±	0.07
18:1 c	0.04	±	0.04
19:1 a	0.01	±	0.01
19:1 b	0.02	±	0.02
20:1ω11c	0.40	±	0.01
20:1ω9c	0.12	±	0
20:1ω7c	0.14	±	0
22:1ω11c	0.05	±	0.04
22:1ω9c	0.10	±	0.07
**PUFA**			
18:3ω6 + 18:5ω3	3.63	±	0.05
18:4ω3	10.44	±	0.13
18:2ω6	3.73	±	0.06
18:3ω3	2.54	±	0.05
20:4ω6	1.09	±	0.01
20:5ω3	13.04	±	0.15
20:3ω6	0.66	±	0.02
20:4ω3	2.93	±	0.03
20:2ω6	0.11	±	0.01
21:5ω3	0.04	±	0.03
22:5ω6	0.16	±	0.02
22:6ω3 DHA	1.70	±	0.02
22:5ω3	0.34	±	0.06
**FALD**			
16:0 FALD	5.11	±	0.06
18:0 FALD	0.26	±	0.04
CLA			
CLAa	0.04	±	0.07
CLAb	0.01	±	0.02
CLAc	0.12	±	0.1
**Sum SFA**	30.73		
**Sum Branched**	1.06		
**Sum MUFA**	22.27		
**Sum PUFA**	40.41		
**Sum FALD**	5.37		
**Sum CLA**	0.17		

Abbreviations: SFA, saturated fatty acid; Branched—branched SFA; MUFA, monounsaturated fatty acid; PUFA, polyunsaturated fatty acid; PA, palmitic acid (16:0); DHA, docosahexaenoic acid (22:6ω3); FALD, fatty aldehyde; CLA, conjugated linoleic acid.

**Table 3:** Production of superoxide anion (pmol O^2−^ cell^−1^ h^−1^) in whole and lysed cells of Chilean *H. akashiwo*. Superoxide from one strain of the Chilean dinoflagellate Karenia selliformis was included for comparative purposes.

Species	Strain	Isolator	Origin	Whole Cells (pmol O^2–^ cell^−1^ h^−1^)		Lysed Cells (pmol O^2–^ cell^−1^ h^−1^)	
				Mean	SD	Mean	SD
*H. akashiwo*	CREAN_HA03	J.I. Mardones, 2019	Chile, Los Lagos	0.308	0.03	0.293	0.013
*K.* *selliformis*	CREAN_KS02	J.I. Mardones, 2018	Chile, Aysén	0.311	0.467	0.861	0.535

## Data Availability

The data presented in this study are available in this article.
